# A high-performance thioredoxin-based scaffold for peptide immunogen construction: proof-of-concept testing with a human papillomavirus epitope

**DOI:** 10.1038/srep04729

**Published:** 2014-04-22

**Authors:** Elena Canali, Angelo Bolchi, Gloria Spagnoli, Hanna Seitz, Ivonne Rubio, Thelma A. Pertinhez, Martin Müller, Simone Ottonello

**Affiliations:** 1 Department of Life Sciences, Biochemistry and Molecular Biology Unit, University of Parma, Italy; 2 German Cancer Research Center, Heidelberg, Germany; 3 C.I.M. Laboratory, Technopole Parma, University of Parma, Italy; 4 These authors contributed equally to this work.

## Abstract

*Escherichia coli* thioredoxin has been previously exploited as a scaffold for the presentation/stabilization of peptide aptamers as well as to confer immunogenicity to peptide epitopes. Here we focused on other key features of thioredoxin that are of general interest for the production of safer and more effective peptide immunogens, such as a high thermal stability, lack of cross-reactivity and a low-cost of production. We identified thioredoxin from the archaebacterium *Pyrococcus furiosus* (PfTrx) as a novel scaffold meeting all the above criteria. PfTrx is a highly thermostable and protease-resistant scaffold with a strong (poly)peptide solubilisation capacity. Anti-PfTrx antibodies did not cross-react with mouse, nor human thioredoxin. Untagged PfTrx bearing a previously identified HPV16-L2 peptide epitope was obtained in a >90% pure form with a one-step thermal purification procedure and effectively elicited the production of neutralizing anti-HPV antibodies. We thus propose PfTrx as a superior, general-purpose scaffold for the construction of safe, stable, and low-cost peptide immunogens.

Reverse vaccinology or vaccinomics, that is the exploitation of genome sequence information from microbial pathogens for the construction of increasingly safe and effective vaccines against human pathogens, is rapidly moving forward thanks to the exponential growth of microbial genomics^1^. In addition to full-length antigens, an important alternative option to fully exploit the potential of reverse vaccinology for the construction of so-called subunit vaccines is the use of immunodominant peptides conjugated to macromolecular carriers as immunogens.

The most common mode of conjugation is random cross-linking of chemically synthesized peptides to scaffold proteins such as ovalbumin and hemocyanin^2^. While quite straightforward, this approach not always provides optimal immunogenicity, especially in the case of weak epitopes, and does not preserve the structural features of the parent antigen region from which the peptide is extracted^2^,^3^. Additional drawbacks in the case of vaccine applications are carrier-induced epitopic suppression^4^ and the intrinsic variability of random cross-linking conjugation^2^,^5^, which may unpredictably affect immunogenicity and preclude batch-to-batch consistency. Various ways to overcome the above limitations have been proposed, including chemically defined conjugation to synthetic immunogenic backbones as in the Multiple Antigenic Peptide (MAP)^6^,^7^,^8^ and lipopeptide^9^ approaches. A remaining hurdle, even with these advanced peptide formulations, is their unsuitability for direct conversion to other vaccine formulations such as virus or human cell transfer for *in vivo* expression and DNA vaccination.

An alternative, recombinant way to overcome most of the above limitations relies on the use of carrier proteins to which various versions of a selected peptide epitope (e.g., single-copy or multicopy, entirely natural or flanked by artificial immunostimulatory sequences) can be joined site specifically in either an end-to-end or an internal fusion configuration^10^. Both approaches can provide chemically defined recombinant antigens that can be used as direct leads for the development and testing of various vaccine formulations. However, the internal fusion setup, although less commonly used, has some additional advantages. These include, for example, a higher resistance to proteolysis^11^,^12^,^13^ and a scaffold-induced constraining that may allow to preserve some of the structural features of the parent antigen^14^. Internal fusion to a variety of scaffold proteins, usually within surface-exposed, structurally stable loops, has been used for a variety of applications besides to recombinant peptide vaccine development^15^,^16^. For example, site-specific exposure of *in vivo* or *in vitro* selectable combinatorial peptide (“aptamer”) repertoires for the construction of artificial “binders” to be used as antibody mimics or protein-protein interaction reagents^16^,^17^,^18^.

One of the best established scaffold proteins is *Escherichia coli* thioredoxin A (EcTrx)^19^. This is a small (109 residues), soluble and non-toxic protein that contains a surface exposed loop (segment 33–36), corresponding to a unique *Cpo*I restriction site in the nucleotide sequence that is suitable for directional in-frame cloning of peptide encoding oligonucleotides, stabilized at the base by a disulfide bond^20^,^19^. This thioredoxin loop has been successfully employed for the intracellular as well as cell surface exposure of peptide aptamers^17^,^21^,^22^. The corresponding EcTrx–peptide aptamer fusion proteins have been used as capture reagents for protein-protein interaction studies (reviewed by Baines^23^) with a performance superior to that of other scaffold proteins^24^. An additional advantage of thioredoxin is its widespread occurrence, which allows the easy retrieval of more or less divergent Trx homologs from a variety of organisms ranging from bacteria to humans.

We have previously exploited the peptide display and constraining capacity of EcTrx for conferring immunogenicity to short (15–19 amino acid residues) tandemly repeated peptides in an approach called Thioredoxin Displayed Multipeptide Immunogens (TDMI). In one case, we conferred strong immunogenicity to a four-fold repeated 15-amino acid peptide derived from the human Aβ peptide and showed that the resulting antibodies preferentially recognized oligomeric and fibrillar Aβ forms, compared to the monomeric form of the Aβ peptide^25^. More recently, we applied the TDMI approach to the human papillomavirus (HPV) minor capsid protein L2^26^. This capsid protein, which contains polypeptide regions conserved among the 15 carcinogenic mucosal HPV types but is poorly immunogenic, is being pursued by various groups as an alternative to the highly immunogenic, but strongly type-specific major capsid protein L1, for the development of a broadly protective HPV prophylactic vaccine^27^,^28^,^29^,^30^,^31^,^32^,^33^. Using TDMI, we scanned the N-terminal (aa. residues 1–120) region of HPV16 L2 and identified a 19 aa L2 peptide (segment 20–38) that upon tandem multimerization within the display site of EcTrx acquired strong immunogenicity and elicited the production of polyclonal^26^ as well as monoclonal^34^ antibodies capable of cross-neutralizing multiple high-risk HPV types.

As shown in a recent study^31^, a monovalent EcTrx-HPV16L2(20–38) prototype immunogen, adjuvanted with Montanide ISA51 and administered to Balb/c mice, compared extremely well in terms of immunogenicity and broad-range HPV neutralization responses with a much more complex to manufacture bivalent L2 vaccine based on adeno-associated virus-like particles (AAVLP). In fact, despite the presence of a monotype epitope, EcTrx-HPV16L2 elicited neutralization titers against all tested viral types (HPV 16, 18, 45, 52, 58 and bovine papillomavirus) except HPV31, that were very similar to those induced by the bivalent AAVLP(HPV16/31L2) vaccine, but with a generally lower variability of response.

Attractive features of a Trx-L2-based vaccine, in addition to a broad cross-neutralization capacity, would be an easy, low-cost production, even in the absence of foreign sequences commonly utilized as tags for affinity purification, and thermal stability. The latter is currently viewed as one of the most relevant and desirable properties of new-generation vaccines, with an expectedly high economic and health impact due to a reduction of vaccine wastage and the prevention of negative health effects caused by the administration of damaged, ineffective vaccines^35^. All the above features would provide important advantages compared to the effective, but highly HPV type-specific, cold-chain requiring and costly L1 virus-like particles (L1-VLPs)-based vaccines (Gardasil^TM^, Merck and Cervarix^TM^, GlaxoSmith-Kline)^36^,^37^,^38^. In fact, although neutralization titers elicited by EcTrx-HPV16-L2 and AAVLP(HPV16/31L2) were 5 to 20 fold lower than those induced by Gardasil and Cervarix against vaccine viral types (HPV16 and 18), both L1-VLP-based vaccines displayed a strikingly narrow and extremely reduced (or absent) cross-neutralization capacity^31^.

In addition to thermal stability and a low-cost of production, another key requisite of a thioredoxin-based vaccine is the lack of cross-reactivity of antibodies generated against the Trx scaffold with host (especially human) thioredoxin. Taking into account all the above potential improvements, we explored the thermal stability and scaffolding capacity of two archaebacterial Trx-like proteins more distantly related to mammalian thioredoxins than EcTrx. We found that a far-divergent thioredoxin-like protein from the archaebacterium *Pyrococcus furiosus* (PfTrx) exhibits a superior thermal stability and solubilization capacity compared to EcTrx, which allowed for the production of nearly-homogeneous, tag-free antigen preparations with an easy and extremely cost-effective one-step thermal purification procedure. We also show that anti-PfTrx antibodies display no cross-reactivity with mammalian thioredoxins.

We thus propose *P. furiosus* thioredoxin as a high-performance, general-purpose scaffold protein for the construction of recombinant peptide immunogens.

## Results

### Identification and preliminary evaluation of alternative archaebacterial thioredoxin scaffold proteins

**Figure 1:**
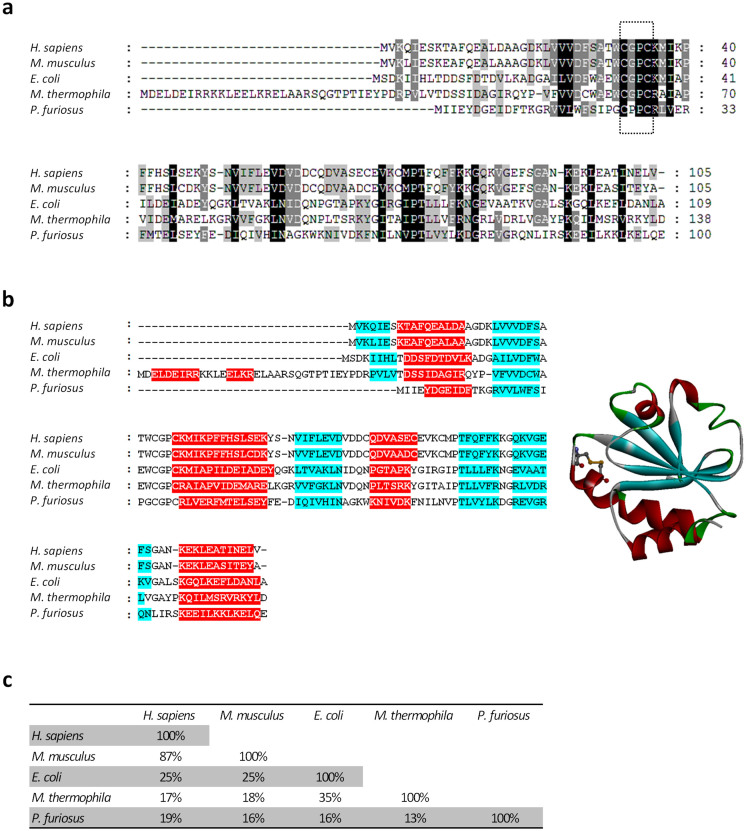
Alignment of putative archaebacterial thioredoxins with *E. coli*, mouse and human thioredoxin. (a) Predicted thioredoxin polypeptides from the archaebacteria *M. thermophila* (Acc. n. YP_843141.1) and *P. furiosus* (Acc. n. NP_578470.1) are aligned with homologous thioredoxins from *E. coli* (Acc. n. YP 003038425), *M. musculus* (Acc. n. NP_035790) and *H. sapiens* (Acc. n. AY004872) (see Supplementary Fig. S1 for an extended phylogeny of bacterial thioredoxins). Amino acids that are identical in all or in three out of the five reported sequences are shown on a *black* or *grey* background, respectively. The conserved active (display) site is boxed. (b) Predicted secondary structures of the five thioredoxin polypeptides; β sheets and α helixes are shown on a *blue* and a *red* background, respectively. The 3D structure of *E. coli* thioredoxin (PDB accession code: 2trx) is shown on the right with the two Cys residues of the redox active/display site represented in a ball and stick format. (c) Amino acid sequence identity values for the five thioredoxin polypeptides addressed in this study.

To identify candidate sequences coding for thioredoxins more distantly related to mammalian thioredoxins and with a higher thermal stability than EcTrx, we used the latter sequence as a query for a BLAST-P search against all sequenced genomes from thermophilic and hyperthermophilic archaeabacteria. Potential homologs (*P* ≤ 10^−3^) were aligned and used to construct a global phylogenetic tree (Supplementary Fig. S1; where EcTrx represents the only eubacterial sequence), which revealed, among different clusters, two major groups: one including EcTrx and other phylogenetically related thioredoxins, and another group mainly comprised of far-divergent thioredoxin-like sequences. Sequences representative of these two groups –one from the thermophile *Methanosaeta thermophila* (optimal growth temperature 55–60°C; accession N. YP_843141.1), the other from the hyperthermophile *Pyrococcus furiosus* (optimal growth temperature ~100°C; accession N. NP_578470.1, hypothetical protein PF0741)– were selected for further analysis. Both predicted polypeptides contain two conserved cysteine residues separated by two amino acids (-CysXXCys-) near to the putative redox-active site (Fig. 1a). They also exhibit a predicted thioredoxin-like secondary structure, with a core β-sheet surrounded by four α-helices (Fig. 1b). Interestingly, *M.* *thermophila* thioredoxin (MtTrx) contains an N-terminal, 30 amino acids extension with two additional predicted α-helices, whereas the Trx-like polypeptide from *P. furiosus* (PfTrx) is nine amino acids shorter at the N-terminus than EcTrx and lacks one predicted N-terminal β-strand which is present in all the other thioredoxins. As shown in Fig. 1c, MtTrx is 35% identical to EcTrx and also contains the conserved Gly-Pro dipeptide interposed between the two Cys residues. A further reduced sequence identity with EcTrx (16%) characterizes the as yet unannotated thioredoxin-like sequence from *P. furiosus*, which also diverges from canonical thioredoxins at the level of the active site sequence (-CysProProCys- instead of –CysGlyProCys-; see Fig. 1). PfTrx was chosen as the lead candidate for our analysis also because of the established utilization of *P. furiosus* as a producer of highly thermostable proteins, including proteins of marked biotechnological interest such as the proof-reading Pfu DNA polymerase^39^. As further shown in Fig. 1c, both selected archaebacterial Trx-like polypeptides, and especially PfTrx, are more distantly related to mouse and human thioredoxins than EcTrx. This suggests that anti-scaffold antibodies elicited by both archaebacterial thioredoxins might be less (or not at all) cross-reactive with mammalian thioredoxins compared to EcTrx (see below).

Codon-optimized *MtTrx* and *PfTrx* synthetic genes (Supplementary Fig. S2), both bearing a unique *Cpo*I site at the nucleotide sequence position corresponding to the active/display site of thioredoxin commonly utilized for the insertion of heterologous peptides, were then designed and used for recombinant expression and 6xHis tag-mediated metal-affinity purification of the corresponding polypeptides. Fluorescence spectroscopy (λ_excitation_ = 280 nm, emission measured between 300 and 450 nm) was initially used to determine the apparent melting temperatures of the two proteins^40^, using His-tagged EcTrx purified in the same way as a reference protein. Temperature was ramped from 25° to 95°C in 5°C steps with a 5 min incubation at each temperature, followed by spectral analysis as a function of temperature. Apparent melting temperatures, calculated as the temperature corresponding to the largest derivative of the sigmoidal curves representing the maximum emission wavelength as a function of temperature, were 80°C and 92°C for EcTrx and MtTrx, respectively. No appreciable spectral change was detected for PfTrx, indicating that *Pyrococcus* thioredoxin did not undergo any appreciable denaturation under these conditions (data not shown).

**Figure 2:**
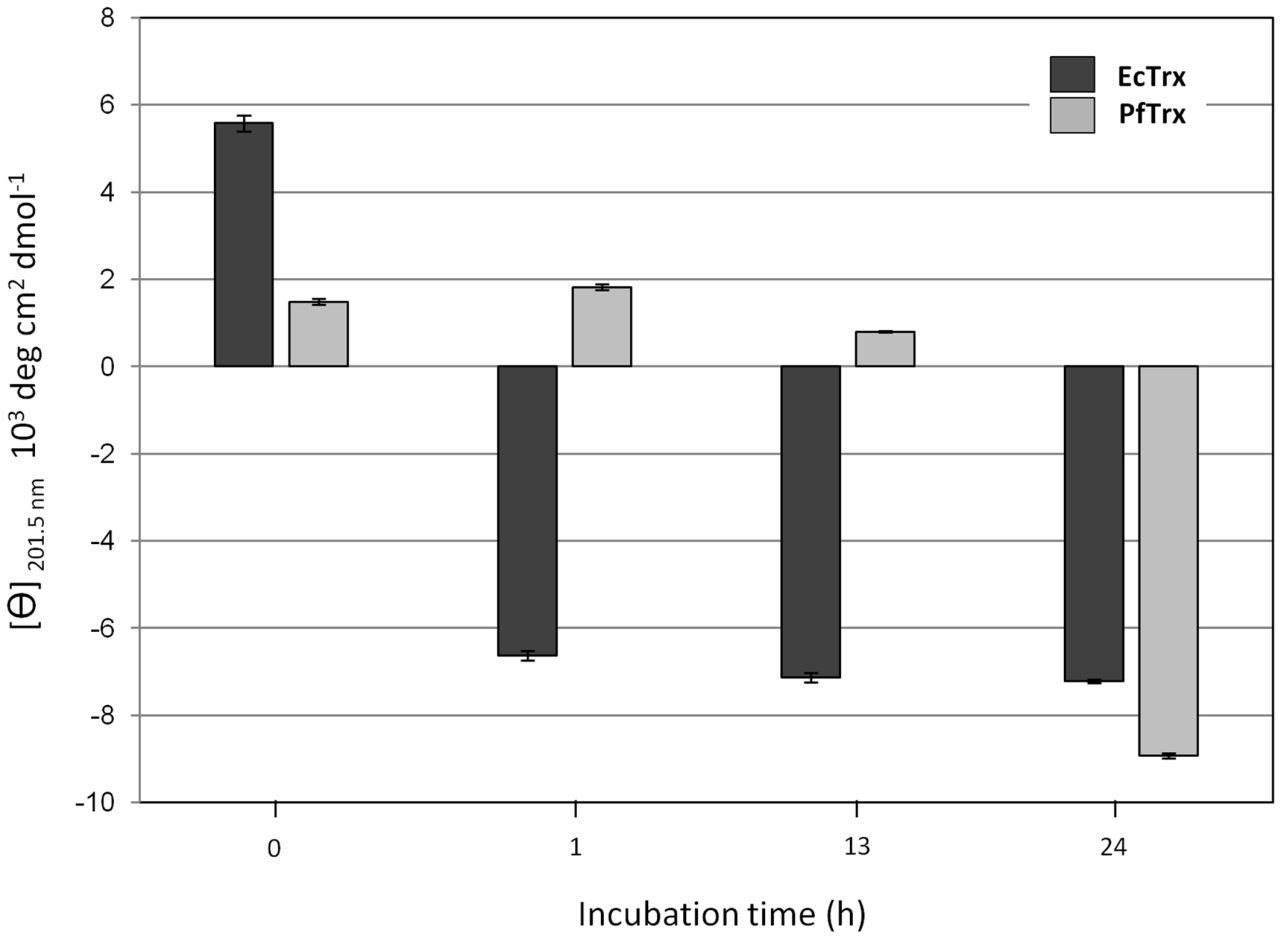
Isothermal denaturation analysis of metal affinity-purified EcTrx and PfTrx. Molar ellipticity at 201.5 nm was measured by circular dichroism on the EcTrx (*black bars*) and PfTrx (*grey bars*) proteins (10 μM in PBS) incubated for the indicated times (0–24 h) at 100°C; positive and negative values indicate a folded or unfolded state, respectively. Data are mean ellipticity values ± s.e.m. from three independent experiments.

Higher temperature conditions (100°C for 1, 13 and 24 h) together with a protein denaturation analysis based on ellipticity (θ) measurements performed by far-UV circular dichroism (CD) spectroscopy were then used to further characterize the three proteins and to gain information on the thermal stability of PfTrx. Surprisingly, even after the shortest incubation time, MtTrx formed insoluble aggregates and nearly all of the protein was recovered as an amorphous precipitate, as if thermal denaturation caused the exposure of hydrophobic patches leading to irreversible aggregation. Under the same conditions (1 h at 100°C) EcTrx was fully denatured, but remained soluble, whereas an additional 23 h were required to fully denature PfTrx (Fig. 2).

Based on the above data, indicating the unfavourable thermal stability properties of MtTrx, we discarded this protein as a potential alternative scaffold. In contrast, empty *Pyrococcus* thioredoxin (i.e., PfTrx without any foreign peptide inserted at the display site) proved to be an exceptionally thermostable protein that was subjected to further analysis.

### Thermal stability, solubilisation capacity and immunogenicity of PfTrx and EcTrx harbouring an HPV16 L2-derived multipeptide epitope

**Figure 3:**
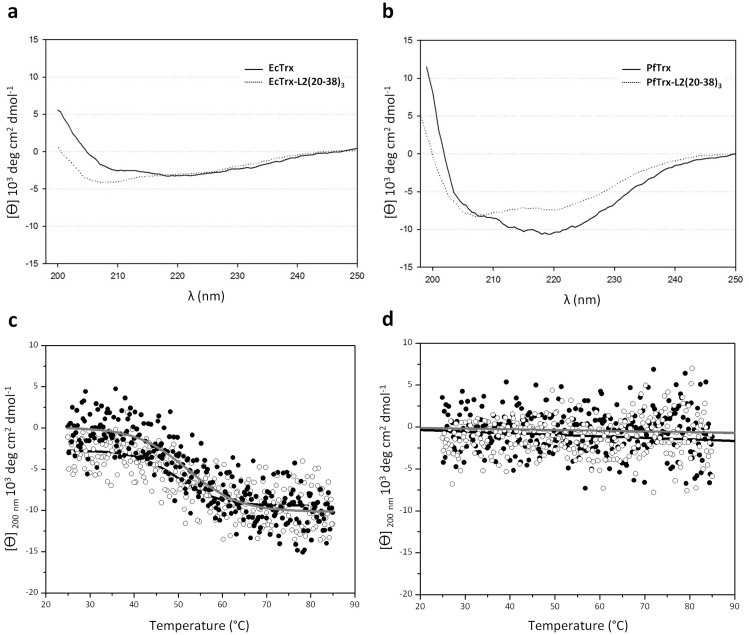
Circular dichroism analysis of empty and L2 tripeptide containing EcTrx and PfTrx. (a) Far-UV CD spectra (recorded at 25°C) of the empty (*solid line*) and the L2 insert-containing (*dotted line*) EcTrx protein (10 μM in PBS) purified by metal-affinity chromatography. (b) Same as (a) for PfTrx. Spectra are given as molar ellipticity [θ] values at different wavelengths (λ). Secondary structure alterations produced by the incorporation of the L2(20-38)_3_ tripeptide are apparent. (c) Far-UV CD analysis of metal affinity-purified EcTrx-L2(20-38)_3_ (10 μM in PBS). Data are presented as variations of molar ellipticity [θ] at 200 nm upon unfolding (25–85°C; *close dots, gray line*) and refolding (85–25°C; *open dots, black line*). Temperature-dependent midpoint transitions at 52.1°C (unfolding) and 49.9°C (refolding) were calculated. (d) Same as in panel C for PfTrx-L2(20-38)_3_, for which no changes of [θ] at 200 nm were observed.

The unique *Cpo*I restriction site previously inserted into the sequence coding for the artificial PfTrx protein variant was used for directional cloning of three tandemly repeated copies of the HPV16 L2(20–38) peptide, with an interposed GlyGlyPro tripeptide spacer added to minimize junctional epitope formation. Following expression and metal-affinity purification, the PfTrx-L2(20–38)_3_ fusion protein and PfTrx without any added insert were analyzed by far-UV circular dichroism at 25°C, using the corresponding EcTrx-based fusion proteins^26^ as references. As shown in Fig. 3 (panels a–b), changes in protein secondary structure attributable to the inserted L2(20–38)_3_ peptide were observed with both L2 insert-bearing fusion proteins. Thermal unfolding of EcTrx-L2(20–38)_3_ (from 25° to 85°C) and its partial refolding (from 85° to 25°C), followed at 200 nm, yielded midpoint transition temperatures of 52.1°C and 49.9°C for unfolding and refolding, respectively (Fig. 3c). Under the same conditions, no significant change was observed with PfTrx-L2(20–38)_3_ (Fig. 3d), indicating a higher thermal stability and perhaps a stronger insert constraining and solubilisation capacity of the PfTrx scaffold compared to EcTrx (see also Fig. 2). Both features might favourably influence peptide epitope presentation and antigen production.

**Figure 4:**
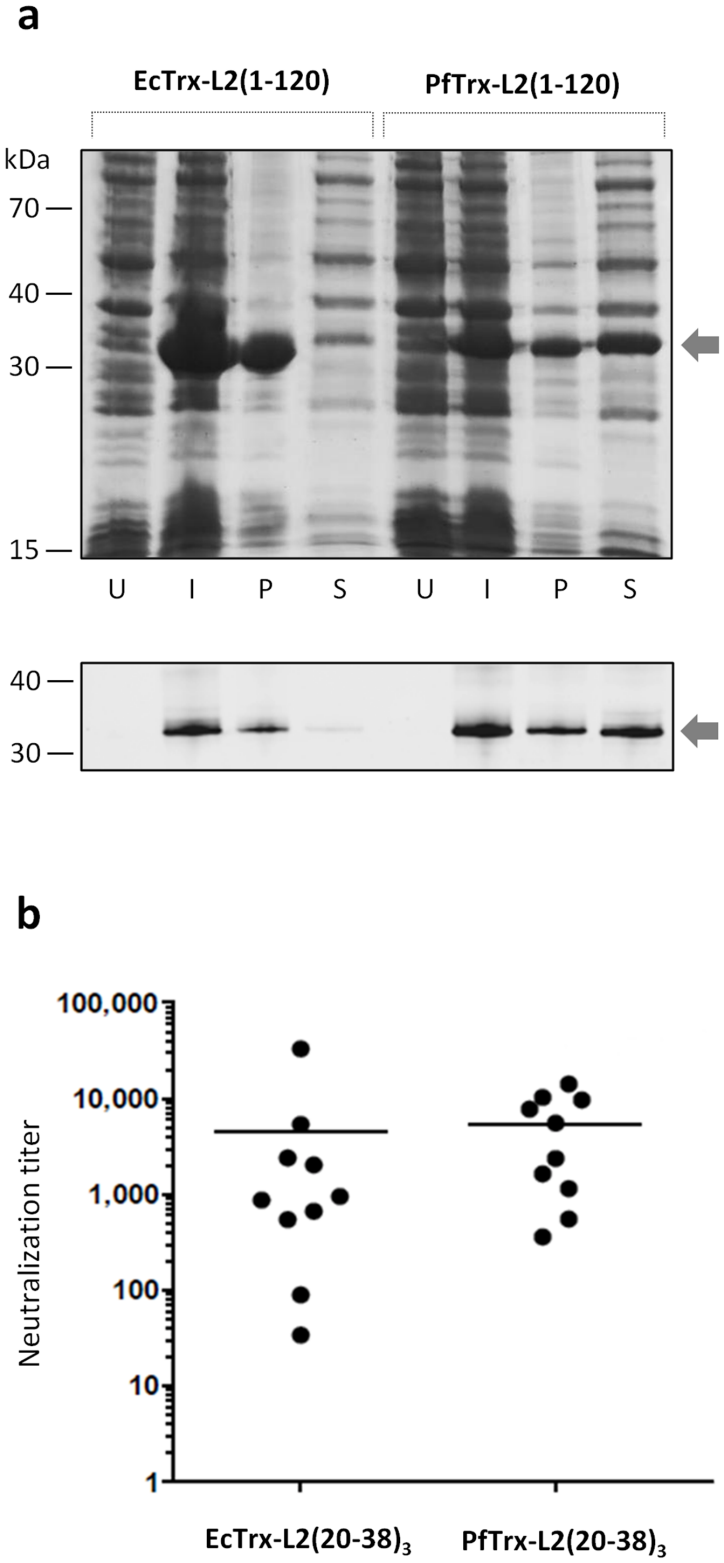
Comparative analysis of the solubilization capacity and immunogenicity of PfTrx and EcTrx. (a) The 1-120 aa. N-terminal region of L2 inserted into the display site of either EcTrx or PfTrx was used to test the solubilisation capacity of the two scaffold proteins. Bacterial cells expressing the two His-tagged Trx-L2(1-120) proteins (*marked with an arrow*) were cultured in parallel (10 ml culture each), transferred to lysis buffer (1 ml), and the resulting lysate (“induced culture”, *I*) was centrifuged in order to separate pellet-associated, insoluble proteins (*P*) from soluble proteins recovered in the supernatant (*S*). An equal volume of the various fractions (15 µl each), including total lysates derived from uninduced bacterial cultures (*U*), was subjected to SDS-PAGE fractionation. A sizeable fraction (>50%) of the PfTrx-L2(1-120) polypeptide was present in the soluble fraction, whereas EcTrx-L2(1-120) was almost completely insoluble (*cf.* lanes 3 and 4 with lanes 7 and 8). The results of an immunoblot analysis performed with an anti-L2(20-38) monoclonal antibody, using scaled-down amounts of the various fractions (0.08 µl equivalents each), are shown below the Coomassie-stained gel image. (b) HPV16 neutralization titers measured in sera collected after the last immunization (3^rd^ boost) from mice immunized with either affinity-purified EcTrx-L2(20-38)_3_ or PfTrx-L2(20-38)_3_ adjuvanted with 50% v/v Montanide ISA720. Dots represent individual neutralization titers, i.e., the maximum dilution of immune sera from the two groups (10 animals each) causing 50% pseudovirion neutralization; geometric means of the titers for each group are indicated by horizontal lines (see ‘Methods' for further details).

Next, we used the entire N-terminal region of HPV16 L2 –a 120 amino acids polypeptide that previously proved to be completely insoluble when expressed as an EcTrx fusion protein and required recovery from inclusion bodies under denaturing conditions^26^– as a test polypeptide to evaluate the solubilisation capacity of *Pyrococcus* thioredoxin. As shown in Fig. 4a, while EcTrx-L2(1-120) was almost completely insoluble and associated to inclusion bodies in the pellet, about 50% of PfTrx-L2(1-120) was found to be soluble and could be recovered from bacterial lysate supernatants without the use of denaturing agents. Similar to EcTrx, MtTrx also failed to solubilize the L2(1–120) polypeptide (data not shown), thus pointing once again to the superior performance of PfTrx as a scaffold protein.

Following up to the above *in vitro* results, we performed an immunization experiment aimed to compare the immunogenicity properties of PfTrx and EcTrx, both bearing three copies of the cross-neutralizing HPV16 L2(20–38) epitope. Two groups of mice were immunized with Montanide ISA720-adjuvanted, metal affinity-purified PfTrx-L2(20–38)_3_ and EcTrx-L2(20–38)_3_, and the resulting immune-sera were used to determine HPV neutralization titers (see ‘Methods'). As shown in Fig. 4b, nearly identical neutralization titers were elicited by both antigens, indicating a similar immunogenic performance of PfTrx- and EcTrx-based L2(20–38)_3_ antigens. However, less variable immune responses were induced by PfTrx-L2(20–38)_3_ and a similar trend was also observed at an earlier stage of immunization (see Fig. S3).

### Immune cross-reactivity of anti-Trx antibodies

**Figure 5:**
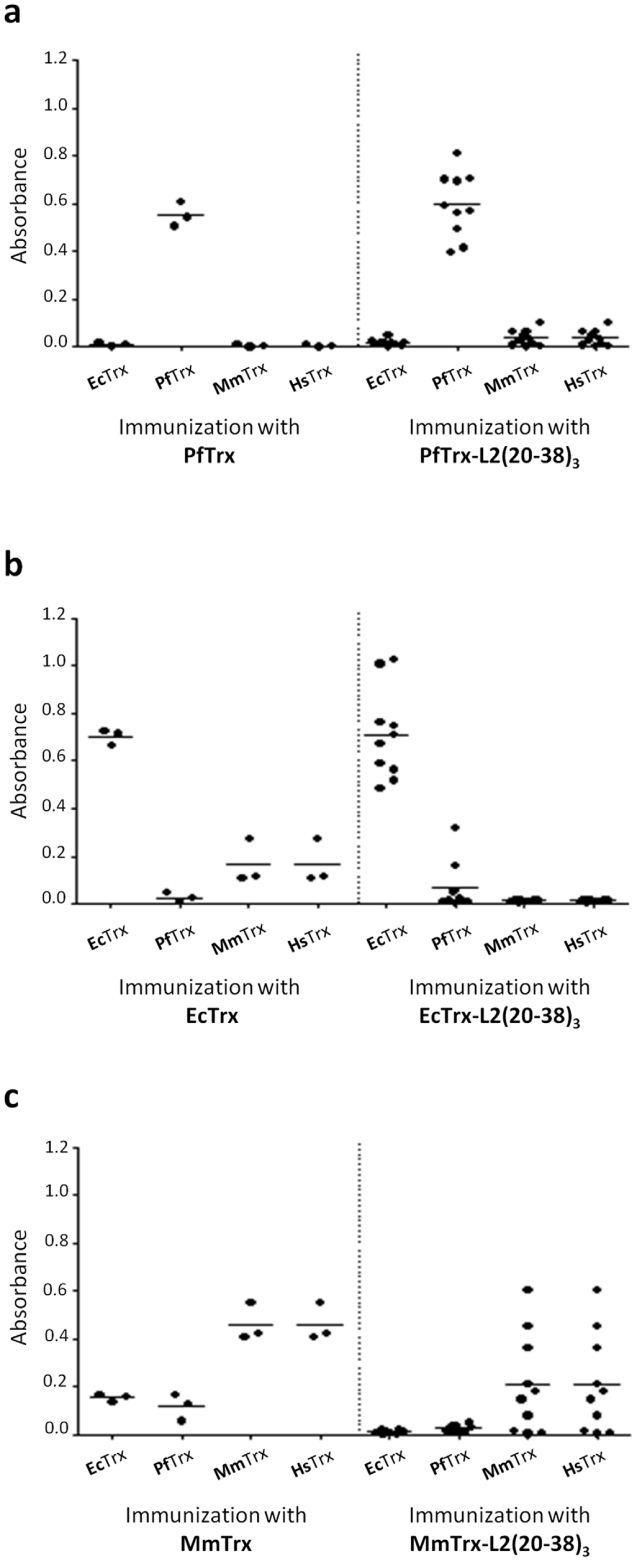
Immune cross-reactivity profiles of anti-PfTrx, anti-EcTrx and anti-MmTrx antibodies. Sera from mice immunized with empty PfTrx, EcTrx and MmTrx (*left graphs* in panels a, b and c, respectively) were analyzed by ELISA using the corresponding GST-fusion proteins plus human thioredoxin (GST-HsTrx) as capture antigens. Data obtained with sera from mice immunized with the corresponding L2(20-38)_3_ insert-containing antigens are shown in the *right graphs* (panels a-c, as above). Each dot represents an individual mouse serum; mean absorbance values measured with each of the four capture antigens are indicated by horizontal lines. The immunization protocol was the same as the one utilized for the experiments reported in Fig. 4b (see ‘Methods' for further details).

His-tagged PfTrx was used as a test antigen for mouse immunization (see ‘Materials and Methods'), with 6xHis EcTrx and mouse thioredoxin (MmTrx) as reference antigens. The anti-Trx (cross)reactivity of the resulting sera was analyzed by ELISA, using PfTrx fused to glutathione S-transferase (GST), together with previously constructed GST-EcTrx, GST-MmTrx and GST-human thioredoxin (HsTrx) fusion proteins, as capture antigens. As expected, maximal immune-reactivity was observed against each of the parent thioredoxin antigens (Fig. 5, *left graphs in each panel*). A significant cross-reactivity with mammalian thioredoxins was detected with sera from mice immunized with EcTrx, which gave above background signals with both MmTrx and HsTrx (Fig. 5b). Similarly, sera from mice immunized with MmTrx, not only cross-reacted with HsTrx, but also showed detectable immune-reactivity with EcTrx and PfTrx (Fig. 5c). In contrast, and in keeping with the extreme sequence divergence of PfTrx from the other thioredoxins (Fig. 1 and Supplementary Fig. S1), sera from mice immunized with PfTrx proved to be the least cross-reactive, with practically no cross-reactivity against mouse or human thioredoxins and a minimal, close to background reactivity with EcTrx (Fig. 5a). Conversely, sera from mice immunized with EcTrx were found to be more cross-reactive with mouse and human thioredoxin than with PfTrx (Fig. 5b); the latter result was independently confirmed by an immunoblot analysis carried out with rabbit anti-EcTrx antibodies that also in this assay displayed no appreciable reactivity with PfTrx (data not shown).

A qualitatively similar picture, albeit with a generally reduced cross-reactivity, was obtained with sera from mice immunized with L2 insert bearing, rather than empty thioredoxins. This included EcTrx, where insert addition completely prevented cross-reactivity against human and mouse thioredoxins (Fig. 5, *right graphs in each panel*). Reduced cross-reactivity upon L2 tripeptide insert addition is likely due to a structural alteration of the Trx active site region utilized for peptide display, which represents the most conserved region (Fig. 1) and probably the major epitope shared by the different thioredoxins.

Overall similar results, pointing again to a low cross-reactivity of antibodies elicited by empty EcTrx (but not PfTrx) with mouse and human thioredoxins were obtained by titration of pooled sera from mice immunized with the two antigens (Fig. S4). Therefore, even though cross-reactivity of anti-EcTrx antibodies with mammalian thioredoxins is significantly quenched by L2 peptide insertion, *Pyrococcus* thioredoxin appears to be the least cross-reactive and potentially most effective scaffold protein. Antibodies elicited by PfTrx-L2 also display an IgG1-skewed isotype distribution with no detectable levels of IgG3 and IgM. This differs from the isotype profile determined in sera from mice immunized with EcTrx-L2 which revealed a dissimilar antibody class switching promoting an increased IgG2a, IgG2b and IgG3 levels (Fig. S5).

### One-step thermal purification and immune performance of a tag-free form of the PfTrxL2(20-38)_3_ antigen

**Figure 6:**
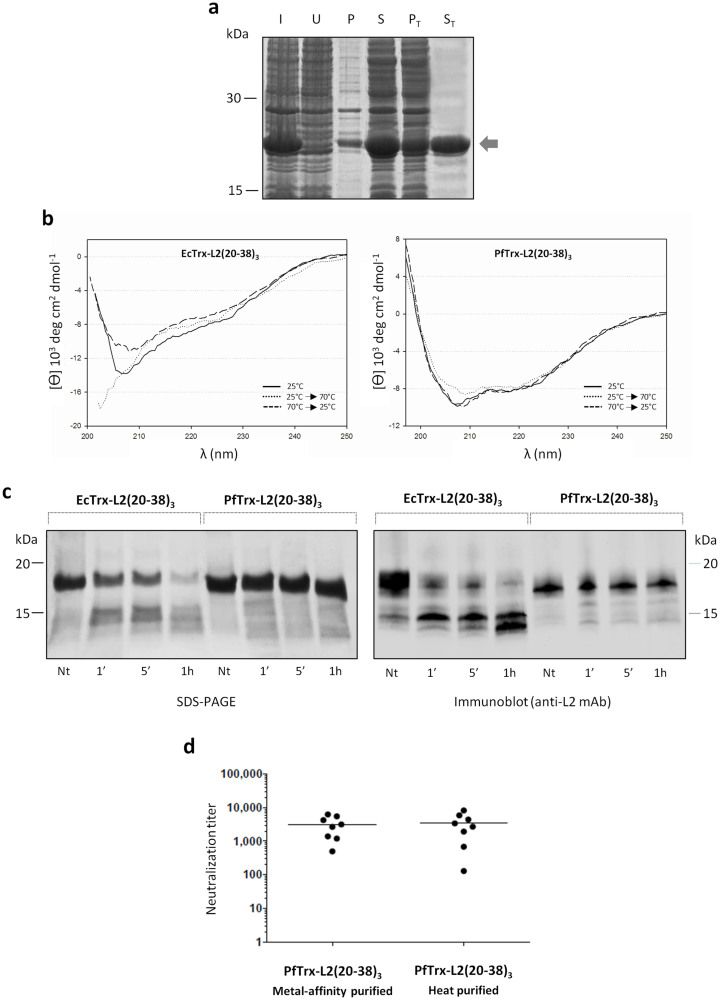
Production and properties of the tag-free, heat-purified form of the PfTrx-L2(20-38)_3_ antigen. (a) SDS-PAGE analysis of PfTrx-L2(20-38)_3_ without His-tag purified with the one-step thermal purification procedure (see ‘Methods' for details). I: induced bacterial culture; U: uninduced culture; P: post-lysis pellet; S: post-lysis supernatant; P_T_: heat-treated post-lysis pellet; S_T_: heat-treated post-lysis supernatant, containing the purified (>90%) PfTrx-L2(20-38)_3_ protein (*marked with an arrow*). (b) Far-UV CD analysis of affinity-purified EcTrx-L2(20-38)_3_ (*left*) and PfTrx-L2(20-38)_3_ (*right*) subjected to the same heat-treatment utilized for thermal purification. Spectra of the unheated proteins (25°C), of the proteins incubated under thermal purification conditions (20 min at 70°C), and of the latter samples returned to 25°C are shown as *solid*, *dotted* and *dashed lines*, respectively. (c) Limited proteolysis analysis. Heat-purified EcTrx-L2(20-38)_3_ and PfTrx-L2(20-38)_3_ were treated for the indicated times with chymotrypsin (enzyme:Trx antigen ratio of 1:100 w/w), fractionated by SDS-PAGE and either stained with Coomassie Blue R-250 (*left* *panel*) or subjected to immunoblot analysis with an anti-L2(20-38) mAb (*right panel*). A sample of each heat-purified Trx-L2(20-38)_3_ protein not treated with chymotrypsin (*Nt*) served as control for these experiments (see ‘Methods' for details). (d) HPV16 neutralization capacity of sera collected after the last immunization from mice immunized with the metal affinity-purified or the heat-purified PfTrx-L2(20-38)_3_ antigen adjuvanted with 50% v/v Montanide ISA720. Dots represent neutralization titers measured in sera from individual immunized animals; geometric means of the titers for the two groups (8 animals/each) are indicated by horizontal lines (see legend of Fig. 4b and ‘Methods' for further details).

Another requirement for the development of human-use approvable recombinant immunogens suitable for phase I clinical investigations is the absence of foreign sequences commonly used for protein purification. We thus explored the feasibility of purifying a His-tag-free form of the PfTrxL2(20-38)_3_ antigen by exploiting its extreme thermal stability (Fig. 3) and high relative abundance in crude soluble lysates from overexpressing recombinant *E. coli* (Fig. 4). To this end, we set up and tested a one-step thermal purification procedure as a means to purify untagged PfTrxL2(20-38)_3_ simply by a 20 min treatment at 70°C, followed by centrifugation to remove heat-denatured and insoluble, contaminating bacterial proteins (see ‘Methods' for details). As shown in Fig. 6a, this procedure allowed us to purify the PfTrx-L2(20-38)_3_ antigen by more than 90% with a yield comparable to that of metal-affinity purification (~20 mg of purified protein/liter of bacterial culture) in less than 1 hour with an extremely low-cost of production.

EcTrx-L2(20-38)_3_ could also be purified by heat-treatment (not shown), but at variance with the PfTrx-L2(20-38)_3_ antigen it required the addition of polyethylene glycol (PEG) 1000 as a protein stabilizer, that had to be removed from the final antigen preparation prior to mice immunization. Also, as revealed by the far-UV CD spectra reported in Fig. 6b, while His-tagged PfTrx-L2(20-38)_3_ pre-purified by metal affinity chromatography and exposed to the same thermal treatment conditions utilized for heat-purification regained exactly the same spectrum of the native, unheated protein, only a partial recovery of the native state CD spectrum was observed in the case of EcTrx-L2(20-38)_3_.

Further evidence as to a generally more compact structure of *Pyrococcus* thioredoxin was provided by limited proteolysis analysis, which revealed a longer-lasting resistance to chymotrypsin of heat-purified PfTrx-L2 compared to the same antigen based on *E. coli* thioredoxin (Fig. 6c).

The *in vivo* immune performance of heat-purified PfTrxL2(20-38)_3_ was then compared with that of a parallel preparation of the His-tagged form of the same antigen purified by metal-affinity chromatography. As shown in Fig. 6d, nearly identical HPV16 neutralization titers were measured in sera from animals immunized with the two antigens, and similar results (not shown) were obtained for cross-neutralization of HPV18 pseudovirions, thus indicating a comparable immunogenicity of the heat- and the metal-affinity purified forms of the PfTrxL2(20-38)_3_ antigen.

Given the possibility that trace amounts of bacterial proteases may contaminate heat-purified preparations thus compromising long-term antigen stability/integrity, we further purified the untagged, heat-purified PfTrxL2(20-38)_3_ antigen on an anion-exchange column and compared the long-term stability of the antigen only subjected to heat-purification with that of the antigen purified by both heat-treatment and anion-exchange chromatography. As shown in Supplementary Fig. S6, the SDS-PAGE profiles of the two antigen preparations remained essentially the same with no sign of protein loss due to proteolytic degradation for up to 7 days at 37°C, both in the presence and in the absence of protease inhibitor supplementation.

## Discussion

We identified *P. furiosus* thioredoxin as a superior protein scaffold for the construction of recombinant peptide immunogens. Valuable features of PfTrx compared to the commonly utilized *E. coli* protein are a higher thermal stability and protease resistance, a greater solubilisation capacity, and the complete lack of cross-reactivity of anti-PfTrx antibodies with other thioredoxins, including human, mouse and *E. coli* Trx.

Using a HPV L2-derived peptide as a specific case study, we exploited the extreme thermal stability of PfTrx to set up a thermal purification procedure that allowed to purify the PfTrx-L2(20-38)_3_ antigen by more than 90% in a single step without the use of foreign sequence tags, nor protein stabilizers. Heat-purified PfTrx-L2(20-38)_3_ was found to be significantly more resistant to chymotrypsin digestion than the corresponding EcTrx antigen and could withstand a 7-days incubation at 37°C without any sign of degradation. HPV neutralization titers elicited by untagged, heat-purified PfTrx-L2(20-38)_3_ were the same as those of the affinity-purified, His-tagged PfTrx and EcTrx antigens. Similar results in terms of immunogenicity were obtained with PfTrxL2(20-38)_3_ stored as a frozen sample and thawed up to three times prior to immunization as well as with the freeze-dried antigen dissolved immediately before immunization (data not shown). Therefore, three basic requirements for an improved, human-use approvable vaccine, namely the lack of cross-reactivity with host proteins, a long shelf-life without the need for cold-chain storage and a low-cost of production in the absence of foreign sequence tags, appear to be fully met by the PfTrx-L2 antigen.

EcTrx-L2 could also tolerate heat purification (in the presence of polyethylene glycol), although it did not fully recover the native state CD spectrum after heat treatment, was less resistant to chymotrypsin proteolysis, and the resulting antibodies were marginally cross-reactive with mammalian thioredoxins. We thus believe that the superior thermal stability and proteolysis resistance, solubilisation capacity and complete lack of cross-reactivity of PfTrx warrant a more straightforward, trouble-free use of this scaffold protein for a variety of peptide antigens and immunogen formulations. The latter may include poorly soluble multipeptide epitopes, longer and/or less stable than the presently tested 57 aa. L2 tripeptide, as well as further functionalization of the thioredoxin scaffold. For example, the incorporation of exogenous universal T-helper epitopes to enhance immunogenicity in particular hosts such as the C57BL/6 mouse strain that was reported to be less responsive to EcTrx-HPV16L2(20-38)_3_ compared to AAVLP(HPV16/31L2)^31^. Other modifications may entail the addition of (poly)peptide molecular adjuvants as well as specific protein modules to anchor the antigen to solid supports in order to produce particulate immunogens. The above modifications may compromise the solubility and/or stability of the resulting conjugates and can thus benefit from the use of an extremely robust protein scaffold.

On a related note, the marked protease resistance of PfTrx may also allow a longer persistence of PfTrx-based antigens at the tissue injection site with a consequently stronger (and more consistent) immune response^41^. This may prove to be particularly important for the development of gastro-protected, orally administrable immunogens that become exposed to the harsh (e.g., protease-rich) environment of the intestine.

Another application of recombinant peptide immunogens, in addition to subunit vaccine construction, is their use for the production of diagnostic polyclonal and monoclonal antibodies. This can also benefit from a robust scaffold with a high solubilization capacity and the ability to tolerate harsh purification conditions. Furthermore, the complete lack of immune cross-reactivity with mammalian thioredoxins would result in a strongly reduced background reactivity of the resulting antibodies.

## Methods

### Trx constructs

Codon-usage optimized *Escherichia coli* (Ec), *Methanosaeta termophila* (Mt), *Pyrococcus furiosus* (Pf) and *Mus musculus* (Mm) *Trx*-coding sequences (see Supplementary Fig. S2) were chemically synthesized (Eurofins MWG Operon) and inserted into the *Nde*I site of a modified pET28 plasmid (Novagen) in frame with two 6xHis tag sequences. The resulting constructs were sequence-verified and transformed into *Escherichia coli* BL21 codon plus (DE3) cells for recombinant protein expression. Glutathione S-transferase (GST) fusions to be used as capture antigens for ELISA analysis were produced from the same sequences, plus *Homo sapiens* (Hs) *Trx*, cloned into the SmaI site of the GST expression vector pGEX-4T-2 Trx-L2(20-38)_3_ antigen constructs containing three tandemly repeated copies of the HPV16-L2 aa 20-38 peptide grafted to the display site of EcTrx and PfTrx as well as the Trx-L2(1-120) constructs were generated by inserting the corresponding sequences into the *Trx* *Cpo*I sites of pre-assembled pET-EcTrx and pET-PfTrx plasmids as described previously^26^. Untagged derivatives of the above constructs were obtained by subcloning the EcTrx-L2(20-38)_3_ and PfTrx-L2(20-38)_3_ inserts, without the dual 6xHis-tag sequences, into the *Nde*I site of the His-tag-lacking pET26 plasmid.

### Expression and purification of the Trx polypeptides

Recombinant protein expression was induced by adding 1 mM isopropyl-β-D-thiogalactopyranoside (IPTG) to the various *E. coli* trasformants, which were then cultured for 3 hours at 37°C. Following cell lysis, 6xHis-tagged polypeptides (both the empty Trx scaffolds and the Trx-L2 fusion polypeptides) were bound to a metal-affinity resin (Talon, Clontech), purified as per the manufacturer's instructions and dialyzed against phosphate-buffered saline (PBS). Untagged Trx-L2 proteins were purified with a modified heat-treatment procedure^42^. Briefly, following bacterial lysate centrifugation, NaCl (0.25 M) was added to the cleared PfTrx-L2(20-38)_3_ supernatant and the resulting solution was incubated for 20 minutes at 70°C, cooled on ice for 10 minutes and centrifuged at 12,000 g for 10 minutes to recover the supernatant containing the purified Trx-L2 protein. A similar procedure, but with the addition of PEG 1000 (12.5%) to the cleared supernatant as stabilizer, was applied to EcTrx-L2(20-38)_3_. GST-Trx fusion proteins were affinity-purified on glutathione-sepharose columns (GE Healthcare) according to the manufacturer's instructions. Trx-L2 antigens to be used for mouse immunization experiments (see below) were detoxified by a 3X Triton X-114 (1% v/v) treatment as described^43^; endotoxin/LPS removal (to levels lower than 2 EU/ml) was verified in each sample by the LAL QLC-1000 test (Lonza). The composition and purity of individual protein preparations were assessed by electrophoretic analysis on 11-15% SDS-polyacrylamide gels and MALDI-TOF analysis. Protein concentration was determined by measurement of absorbance at 280 nm using calculated extinction coefficients and with the use of a Qubit® 2.0 Fluorometer (Life Technologies).

### Melting temperature measurements by fluorescence spectroscopy

Fluorescence measurements were performed with a Jasco FP-6200 Spectrofluorimeter equipped with a Peltier temperature controller, using a fixed 10 μM protein concentration in PBS. Thermal unfolding was carried out in the 25-95°C temperature range with 5°C temperature increments, and samples were allowed to equilibrate for 5 min at each temperature before spectral measurements. Tryptophan and tyrosine residues were excited at 280 nm and fluorescence emission was measured between 300 and 450 nm with a bandwidth of 5 nm, a data-pitch of 1 nm and a scanning speed of 60 nm/min.

### Far-UV circular dichroism spectroscopic analyses

Far-UV CD spectra (190–260 nm) were acquired with a Jasco J715 Spectropolarimeter equipped with a Peltier temperature controller, using a 0.1 cm path-length cuvette, a bandwidth of 1 nm, a data pitch of 0.5 nm, and a response time of 4 s; CD spectra were averaged from 4 scans. Protein concentration was 10 μM in PBS for the single spectra recorded at 25°C and 0.1X PBS for thermal stability measurements. Following baseline correction, the measured ellipticity, θ (mdeg), was converted to the molar mean residue ellipticity [θ] (deg.cm^2^.dmol^−1^), using[θ] = θ/10 cn_res_*l*, where θ is ellipticity, c is the molar concentration of the protein, n_res_ is the number of amino acid residues in the protein and *l* is the optical path length in centimeters.

Thermal unfolding/refolding studies were performed in the 25°C–85°C temperature range; temperature scan was 1°C/min in order to maintain thermal equilibrium. The loss of secondary structure was followed by measuring [θ] at 200 nm. Tm values were calculated as the inflection point of unfolding curves fitted according to the single-step (two-state) transition model.

### Immunoblot analyses

Whole cell extracts or purified proteins were electrophoresed on Criterion TGX precast 4–20% gels (Bio-Rad) in Tris/Glycine buffer. Fractionated samples were electro-transferred to 0.2 µm nitrocellulose membranes (Bio-Rad), that were blocked with 5% skin milk in Tris-buffered saline at 4°C for 12 h. Membranes were then washed with the same buffer and incubated with the anti-L2(20-38) monoclonal antibody K4^34^ (diluted 1:2000) at room temperature for 1 h, followed by the addition of an IRDye 680-conjugated, goat anti-mouse secondary antibody (diluted 1:15000) and visualization of immune-reactive bands by near infrared fluorescence (NIRF) with an Odissey (LI-COR) imager.

### Limited proteolysis

Heat-purified EcTrx-L2(20-38)_3_ and PfTrx-L2(20-38)_3_ (5 µg each) were incubated with 0.05 µg of chymotrypsin (Sigma-Aldrich; enzyme: protein ratio of 1:100 w/w) at 37°C for different lengths of time (from 1 min to 1 hour). Reactions, carried out in 50 mM Tris-HCl pH 8.0, 100 mM NaCl, were stopped by adding SDS-containing electrophoresis sample buffer and analyzed by SDS-PAGE (12% acrylamide gels).

### Mouse immunization and pseudovirion-based neutralization assays

Procedures in animal experiments were approved by the Regierungspräsidium Karlsruhe under permit G246/11 and G19/13. Six- to eight-weeks-old female BALB/c mice (Charles River; Sulzfeld, Germany) were kept in the animal house facility of the German Cancer Research Center under pathogen-free conditions, in compliance with the regulations of the Germany Animal Protection Law. Mice were immunized subcutaneously four times at biweekly intervals with 15 μg of the various detoxified and filter-sterilized Trx-L2 antigens adjuvanted with 50% (v/v) Montanide ISA 720 (Seppic, France). Eight weeks after the last immunization, blood samples were collected by cardiac puncture.

Pseudovirion preparation and neutralization assays were performed as described^44^. Briefly, 50 μl of serially diluted immune sera (or control monoclonal antibodies) were mixed with 50 μl of diluted pseudovirion stocks and incubated at room temperature for 30 min. Next, 50 μl of HeLaT cells (2.5 × 10^5^ cells/ml) were added to the pseudovirion-antibody mixture and incubated for 48 h at 37°C under a 5% CO_2_ atmosphere. The amount of secreted Gaussia luciferase was then determined in 10 μl of cell culture medium using coelenterazine substrate and Gaussia glow juice (PJK, Germany) according to the manufacturer's instructions. A microplate luminometer (Victor3, PerkinElmer) was used to measure luminescence in the culture medium 15 min after substrate addition.

### Immune cross-reactivity analyses

GST–Trx capture ELISAs were carried out in 96-well plates pre-coated with glutathione-casein and subsequently blocked with casein buffer as described previously^45^. Glutathione-casein-coated plates were incubated for 1 h at 37°C with cleared lysates (diluted to 0.5 mg/ml total protein) containing the different GST–Trx fusion proteins. This was followed by the addition of casein blocking buffer and three washes with Tween-20 (0.3% v/v)-PBS; a GST alone (unfused protein) lysate served as a negative control for these assays. Sera from mice immunized with either EcTrx, PfTrx and MmTrx, with and without the L2 tripeptide insert (10 and 3 animals/group, respectively), were analysed both individually at a fixed 1:300 dilution and by serial dilutions of pooled sera from different immunization groups. Following serum addition and incubation, plates were washed three times as above and incubated with a horseradish peroxidase-conjugated goat anti-mouse secondary antibody (Dianova) previously diluted 1:3000 in 1.5% milk-PBS plus 0.3% Tween-20. Plates were incubated for 1 h at 37°C, washed three times, and developed by adding the ABTS [2,2′-azino-bis(3-ethylbenz-thiazoline-6-sulfonic acid)] staining solution (1 mg/ml in 100 mM sodium acetate-phosphate buffer pH 4.2, plus 0.015% H_2_O_2_; 100 μl/well). Absorbance at 405 nm was measured after 40–60 minutes with an automated plate reader (Titertek, Berthold).

### Immunoglobulin isotyping

Immunoglobulin subclasses in sera from mice immunized with the different thioredoxin scaffolds were determined by ELISA using a mouse antibody isotyping kit (BD Pharmingen). Briefly, 96-well plates were coated overnight at 4°C with individual isotype-specific (IgG1, IgG2a, IgG2b, IgG3, IgM, IgA) rat anti-mouse monoclonal antibodies (mAb) in 1X PBS. After washing with 0.05% Tween-20 in PBS, plates were blocked with 1% BSA in 1X PBS and incubated at room temperature for 30 minutes. Ten sera for each group of mice immunized with the different Trx antigens (PfTrx-L2(20-38)_3_, EcTrx-L2(20-38)_3_ and MmTrx-L2(20-38)_3_) were pooled and an aliquot of each pool was added to the wells at increasing serial dilutions, starting from a 1:5000 dilution. Following incubation at room temperature for 1 h, the plates were washed three times as above and incubated with a HRP-conjugated, rat anti-mouse immunoglobulin mAb previously diluted 1:100 in PBS containing 1% BSA for 1 h at room temperature. After three washes, the reaction was developed by adding the HRP substrate solution and absorbance at 450 nm was read after 10 minutes with a microtiter plate reader (Bio-Rad).

### Bioinformatic and statistical analysis

Sequence alignments were performed with ClustalW and imported into the Molecular Evolutionary Genetics Analysis package (MEGA 4.1)^46^; percentage identity was calculated and visualized with the GeneDoc program (http://www.psc.edu/biomed/genedoc/). Secondary structures were predicted with the YASPIN program (http://www.ibi.vu.nl/programs/yaspinwww/). Statistical significance of neutralization assay results was determined with the non-parametric Mann-Whitney-Wilcoxon test performed with GraphPad Prism 5.00 (GraphPad Software, USA); differences between groups were considered significant at *p* < 0.05.

## Author Contributions

E.C. and A.B., who are co-first authors, conducted bioinformatic, protease resistance and long-term stability analyses and produced all the recombinant proteins. G.S. and T.A.P. performed spectroscopic analyses. H.S. and I.R. took care of mice immunization experiments, HPV neutralization assays and immune cross-reactivity analyses. M.M. and S.O. designed the study and supervised all the experiments; S.O. wrote the paper. All authors discussed the results and their implications and commented on the manuscript at all stages of this work.

## Supplementary Material

Supplementary Information Supplementary figures
