# Supplementary material for: A high-performance thioredoxin-based scaffold for peptide immunogen construction: proof-of-concept testing with a human papillomavirus epitope
Source: Sci Rep. 2014 Apr 22;4:4729. doi: 10.1038/srep04729 (PMC3994442; doi:10.1038/srep04729)
Supplement: Supplementary Information — Supplementary figures [file srep04729-s1.pdf]

A high-performance thioredoxin-based scaffold for peptide immunogen construction: proof-of-concept testing with a human papillomavirus epitope

Elena Canali, Angelo Bolchi, Gloria Spagnoli, Hanna Seitz, Ivonne Rubio, Thelma A. Pertinhez, Martin Müller and Simone Ottonello

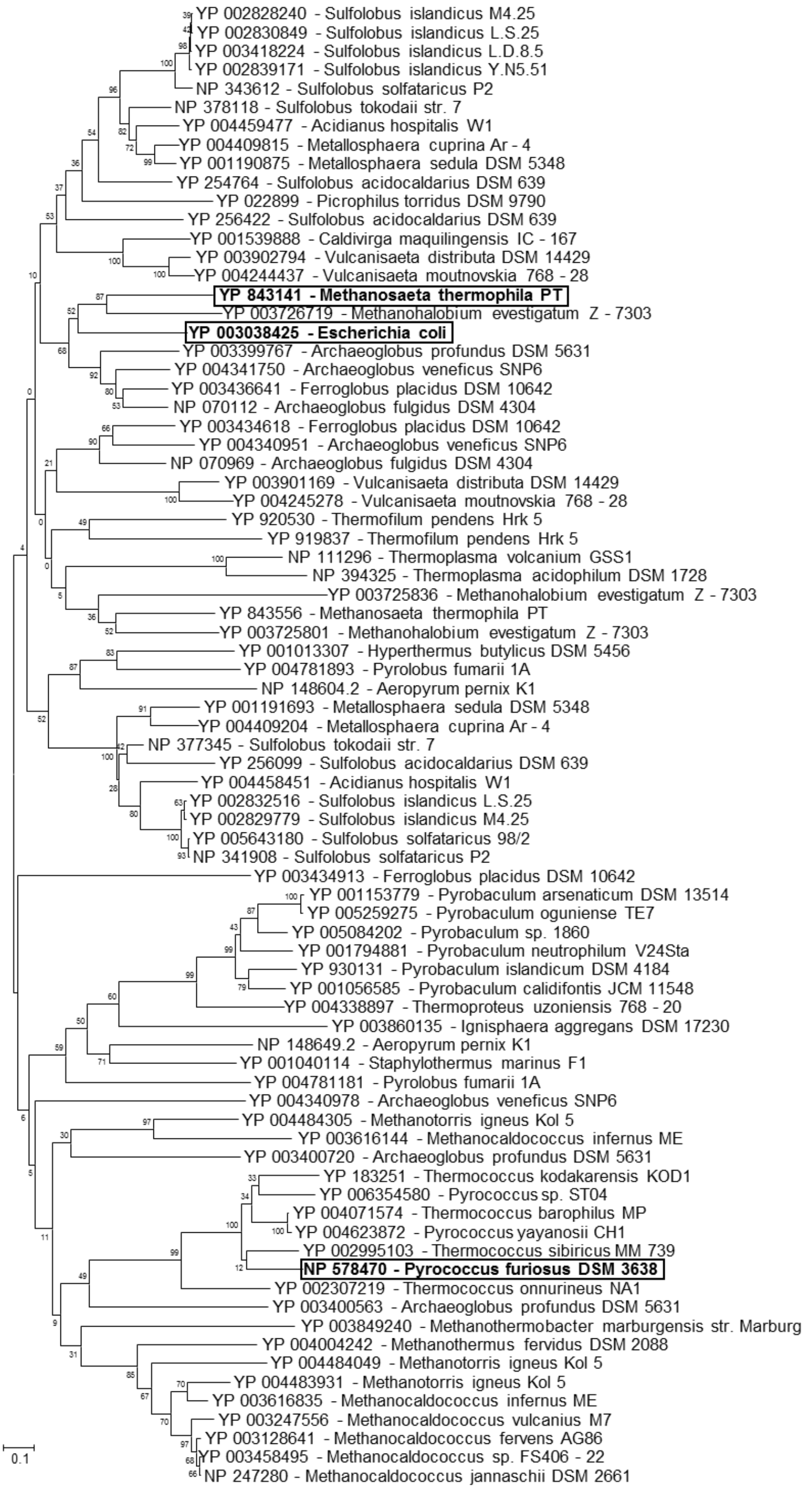

Supplementary Figure S1. Phylogenetic tree of thermophilic and hyperthermophilic archaebacterial thioredoxins using EcTrx as a eubacterial reference. *E. coli* thioredoxin (EcTrx) was aligned with homologous polypeptides (annotated as 'putative thioredoxin' as well as unannotated sequences) from thermophilic and hyperthermophilic archaebacteria. Aligned sequences were used for phylogenetic tree construction using neighbor-joining. Accession number and organism source are shown for each sequence. Bootstrap values for each node are indicated; branch lengths correspond to genetic distances. Thioredoxins from *M. thermophila, E. coli* and *P. furiosus* are boxed.

EcTrx

ATGGGCGATAAAATTATTCACCTGACTGACGACAGTTTTGACACGGATGTACTCAAAGCGGACGGGGCGA
TCCTCGTCGATTTCTGGGCAGAGTGGTG**CGGTCCG**TGCAAAATGATCGCCCCGATTCTGGATGAAATCGC
TGACGAATATCAGGGCAAACTGACCGTTGCAAAACTGAACATCGATCAAAACCCTGGCACTGCGCCGAAA
TATGGCATCCGTGGTATCCCGACTCTGCTGCTGTTCAAAAACGGTGAAGTGGCGGCAACCAAAGTGGGTG
CACTGTCTAAAGGTCAGTTGAAAGAGTTCCTCGACGCTAACCTGGCGTGA

MtTrx

ATGGACGAGCTGGACGAAATCCGCCGTAAAAAACTGGAAGAACTGAAACGTGAACTGGCTGCCCGTAGTC
AAGGAACACCGACGATCGAGTATCCTGACCGCCCTGTACTGGTTACTGATTCTAGCATTGATGCCGGGAT
CCGCCAATATCCTGTCTTTGTGGTGGACTGTTGGGCTGAATGGTG**CGGTCCG**TGTCGTGCTATTGCTCCG
GTGATCGATGAAATGGCCCGTGAGCTGAAAGGACGTGTGGTATTCGGGAAACTGAACGTGGACCAAAATC
CGCTGACGAGTCGTAAATATGGCATTACCGCCATCCCTACACTGCTGGTTTTCCGTAACGGTCGTCTGGT
TGATCGCCTGGTTGGTGCTTATCCGAAACAAATTCTGATGTCTCGTGTCCGTAAATATCTGGACTAG

PfTrx

ATGATTATCGAGTATGACGGCGAAATCGACTTCACCAAAGGTCGTGTTGTACTGTGGTTTAGCATTCCGG
GATG**CGGTCCG**TGTCGTCTGGTTGAACGCTTCATGACCGAACTGAGCGAGTATTTTGAGGATATCCAAAT
TGTCCATATCAATGCCGGCAAATGGAAAAACATCGTAGACAAATTCAATATTCTGAACGTGCCGACCCTG
GTATATCTGAAAGATGGCCGTGAGGTTGGACGCCAAAACCTGATTCGTTCTAAAGAAGAGATTCTGAAAA
AACTGAAAGAGCTGCAGGAGTAA

MmTrx

ATGGTGAAGCTGATCGAGAGCAAGGAAGCTTTTCAGGAGGCCCTGGCCGCCGCGGGAGACAAGCTTGTCG
TGGTGGACTTCTCTGCTACGTGGTG**CGGTCCG**TGCAAAATGATCAAGCCCTTCTTCCATTCCCTCTGTGA
CAAGTATTCCAATGTGGTGTTCCTTGAAGTGGATGTGGATGACTGCCAGGATGTTGCTGCAGACTGTGAA
GTCAAATGCATGCCGACCTTCCAGTTTTATAAAAAGGGTCAAAAGGTGGGGGAGTTCTCCGGTGCTAACA
AGGAAAAGCTTGAAGCCTCTATTACTGAATATGCCTAA

HsTrx

ATGGTGAAGCAGATCGAGAGCAAGACTGCTTTTCAGGAAGCCTTGGACGCTGCAGGTGATAAACTTGTAG
TAGTTGACTTCTCAGCCACGTGGTGTGGGCCTTGCAAAATGATCAAGCCTTTCTTTCATTCCCTCTCTGA
AAAGTATTCCAACGTGATATTCCTTGAAGTAGATGTGGATGACTGTCAGGATGTTGCTTCAGAGTGTGAA
GTCAAATGCATGCCAACATTCCAGTTTTTTAAGAAGGGACAAAAGGTGGGTGAATTTTCTGGAGCCAATA
AGGAAAAGCTTGAAGCCACCATTAATGAATTAGTCTAA

Supplementary Fig. S2. Thioredoxin gene sequences utilized in this work. *EcTrx* and *HsTrx* are the natural gene sequences. *MtTrx* and *PfTrx* are synthetic genes optimized for *E.coli* codon usage and with an added *Cpo*I restriction site in the sequence region corresponding to the display site; the natural display site sequence of *PfTrx* (CPPC) was converted into the canonical CGPC sequence. *MmTrx* is the natural mouse thioredoxin gene with an added *Cpo*I restriction site introduced within the display site sequence by PCR mutagenesis. *Cpo*I restriction sites are in bold and underlined.

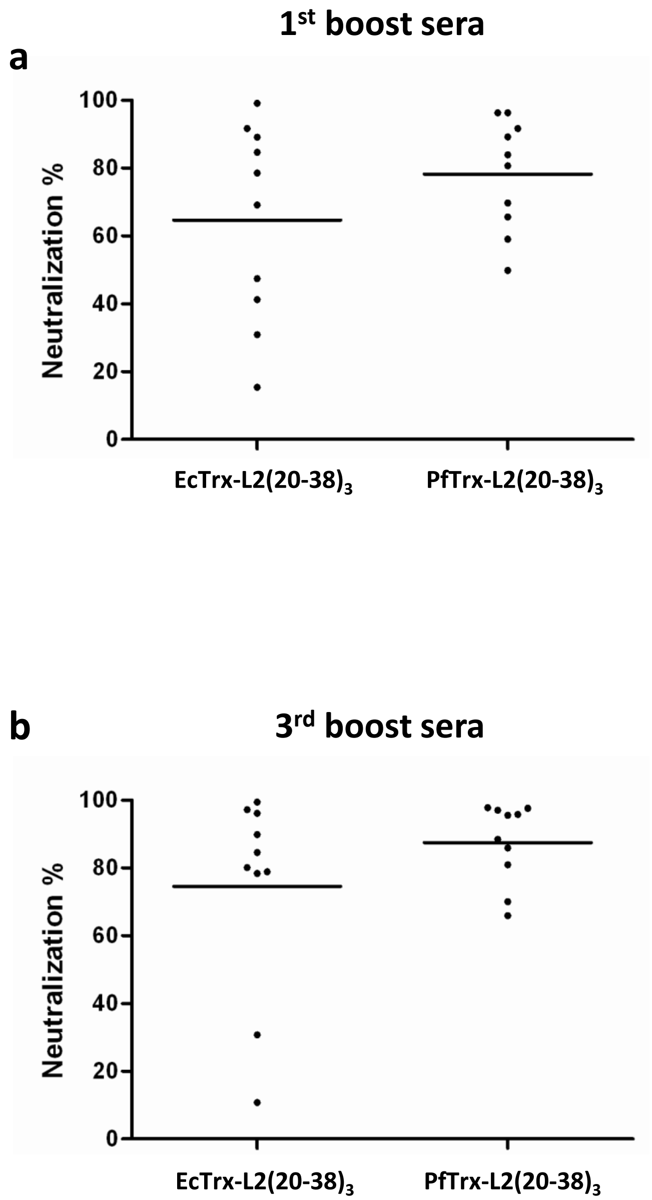

Supplementary Fig. S3. HPV16 neutralization capacity of intermediate sera from mice immunized with His-tagged EcTrx-L2(20-38)3 and PfTrx-L2(20-38)3 antigens. (a) HPV16 neutralization capacity of individual sera collected after the 1st boost immunization with the indicated Trx-L2 antigens was assayed at a fixed 1:200 dilution. Data are expressed as relative neutralization values with respect to the complete pseudovirion neutralization achieved with a reference antibody (mean neutralization percentages are indicated); dots represent individual mouse sera. Similar data for the same set of sera collected after the last immunization (3rd boost) are shown for comparison in panel (b).

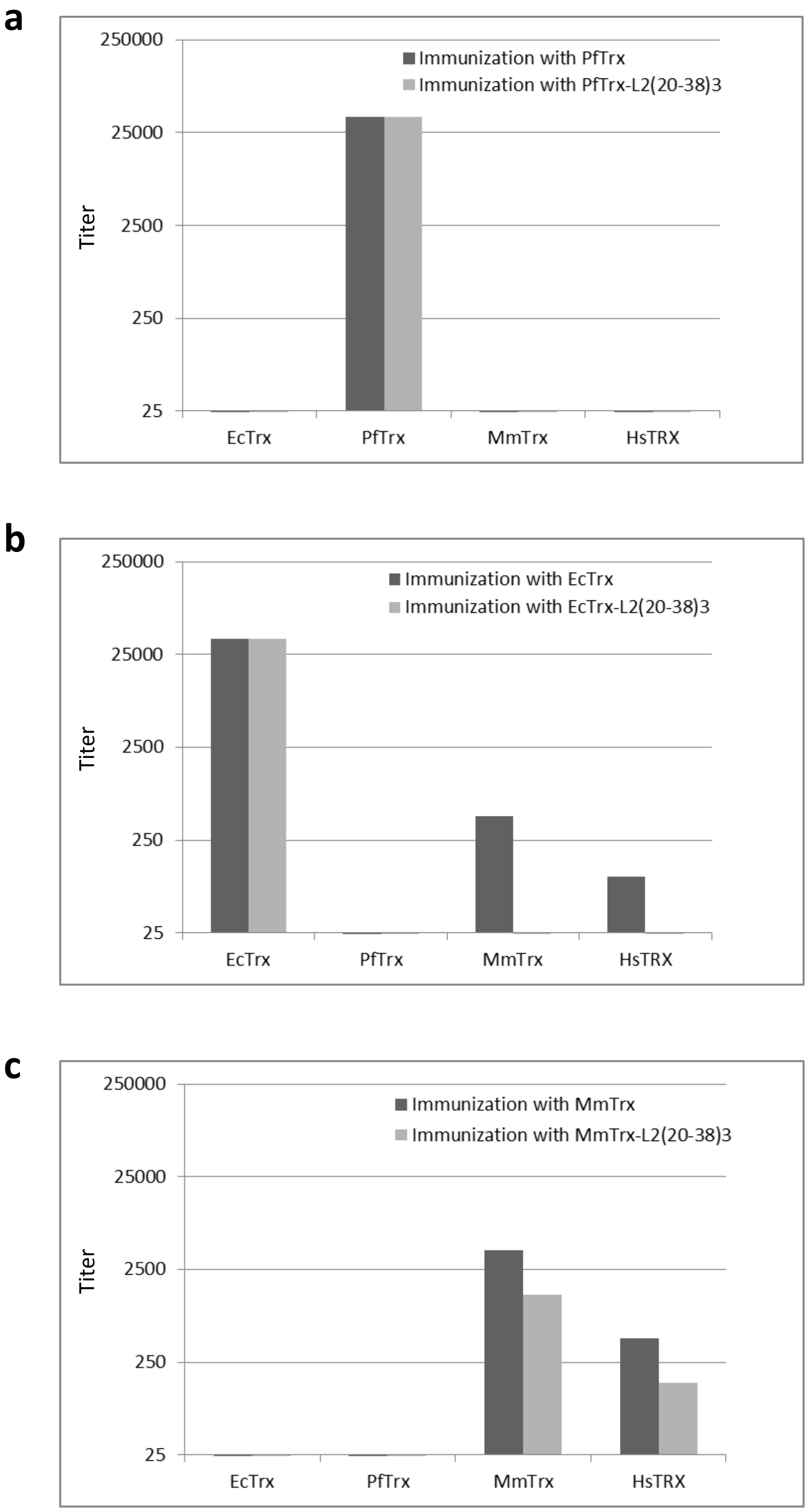

Supplementary Fig. S4. **Titration of antibodies directed against different Trx proteins in pooled sera from mice immunized with the empty scaffold proteins (PfTrx, EcTrx, MmTrx) and the corresponding proteins bearing the L2(20-38)$_3$ tripeptide.** GST-Trx ELISAs and serial dilutions of the indicated sera (collected after the third boost) were used to determine anti-Trx antibody titers; the GST-Trx types utilized as capture antigens are indicated on the *x*-axis.

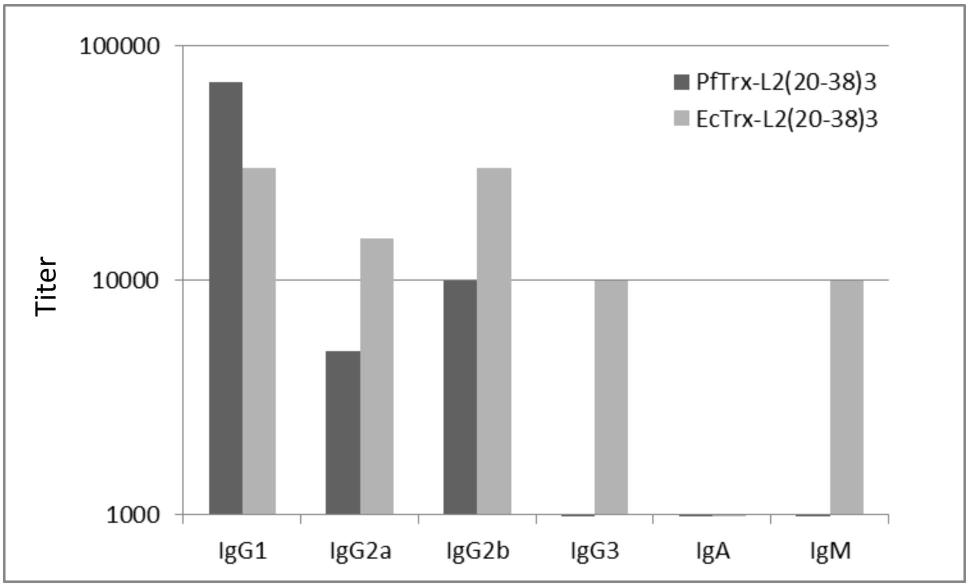

Supplementary Fig. S5. Isotype distribution of antibodies in sera from mice immunized with PfTrx-L2 and EcTrx-L2 antigens. The bar plot shows the titers of the indicated immunoglobulin types in pooled sera from mice immunized with the two antigens; sera were collected after the last immunization (3rd boost).

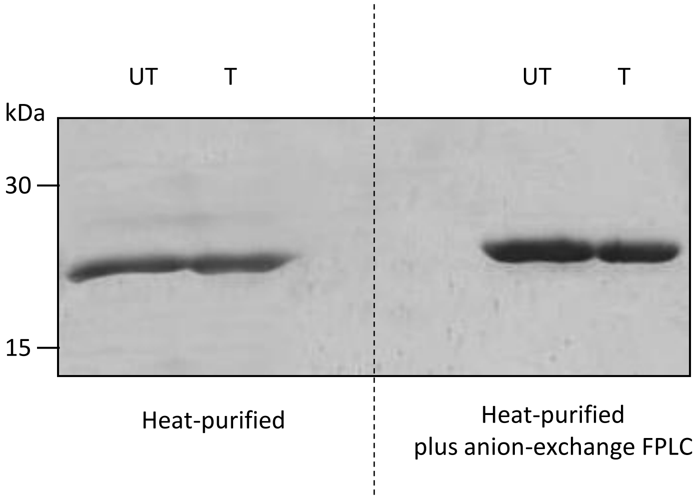

Supplementary Fig. S6. Long-term stability of heat-purified PfTrx-L2(20-38)$_3$. PfTrx-L2(20-38)$_3$ purified by heat treatment and an aliquot of the same protein preparation further purified by anion exchange FPLC were incubated for 7 days at 37°C (treated, T) and their integrity, analyzed by SDS-PAGE, was compared with that of the corresponding proteins kept at –80°C (untreated; UT).
